# Genetic parameter estimates for daily predicted gross feed efficiency and its association with energy-corrected milk in South African Holstein cattle

**DOI:** 10.1007/s11250-023-03741-x

**Published:** 2023-09-28

**Authors:** Matome A. Madilindi, Oliver T. Zishiri, Bekezela Dube, Cuthbert B. Banga

**Affiliations:** 1 Discipline of Genetics, College of Agriculture, Engineering and Science, University of KwaZulu-Natal, Private Bag X54001, Durban, 4000 South Africa; 2 ARC-Animal Production, Private Bag X2, Irene, 0062 South Africa; 3 Department of Animal Sciences, Faculty of Science, Tshwane University of Technology, Private Bag X680, Pretoria, 0001 South Africa; 4 Department of Agriculture and Animal Health, University of South Africa, Private Bag X6, Florida, 1710 South Africa; 5 Department of Animal Sciences, Faculty of Animal and Veterinary Sciences, Botswana University of Agriculture and Natural Resources, Private Bag 0027, Gaborone, Botswana

**Keywords:** Predicted feed efficiency, Genetic improvement, Lactation stage, Repeatability model

## Abstract

Genetic parameters for daily predicted gross feed efficiency (pGFE) and energy corrected milk (ECM) in the first three parities of South African Holstein cattle were estimated by repeatability animal models. Data comprised of 11,068 test-day milk production records of 1,575 Holstein cows that calved between 2009 and 2019. Heritability estimates for pGFE were 0.12 ± 0.06, 0.09 ± 0.04 and 0.18 ± 0.05 in early, mid and late lactation, respectively. Estimates were moderate for primiparous (0.21 ± 0.05) and low for multiparous (0.10 ± 0.04) cows. Heritability and repeatability across all lactations were 0.14 ± 0.03 and 0.37 ± 0.03, respectively. Genetic correlations between pGFE in different stages of lactation ranged from 0.87 ± 0.24 (early and mid) to 0.97 ± 0.28 (early and late), while a strong genetic correlation (0.90 ± 0.03) was found between pGFE and ECM, across all lactations. The low to moderate heritability estimates for pGFE suggest potential for genetic improvement of the trait through selection, albeit with a modest accuracy of selection. The high genetic correlation of pGFE with ECM may, however, assist to improve accuracy of selection for feed efficiency by including both traits in multi-trait analyses. These genetic parameters may be used to estimate breeding values for pGFE, which will enable the trait to be incorporated in the breeding objective for South African Holstein cattle.

## Introduction

The dairy industry is increasingly under pressure to improve feed efficiency, due to the need to maintain herd profitability in an era of increasing feed costs, as well as growing concerns to safeguard the environment (Connor 2015; Miglior et al. 2017; Løvendahl et al. 2018; Krattenmacher et al. 2019; Madilindi et al. 2022a). Gross feed efficiency (GFE) is an important trait in dairy production that provides valuable information about the efficiency of lactating cows to utilise feed. It is measured as the ratio between kilograms of milk or energy-corrected milk produced and kilograms of dry matter intake (DMI) (Chesnais et al. 2016).

Dry matter intake is a major component of feed efficiency traits, including GFE (Connor, 2015; Chesnais et al. 2016; Madilindi et al. 2022a). Direct measurement of DMI from individual lactating cows is generally difficult, and may be achievable only in research stations or appropriately-equipped commercial herds, under a total mixed ration-feeding system (Manzanilla-Pech et al. 2014; de Haas et al. 2015; Li et al. 2018; Krattenmacher et al. 2019). It is thus difficult to obtain DMI data directly from lactating cows on a large scale (Miglior et al. 2017; Madilindi et al. 2022a), which presents a serious challenge to selection for feed efficiency.

Due to scarcity of data on feed intake, there is a paucity of information on the genetic attributes of GFE in dairy cows (Spurlock et al. 2012; Köck et al. 2018). A study by Spurlock et al. (2012) described GFE as being moderately heritable in American Holstein cows, with estimates of 0.20 ± 0.12 in mid lactation and 0.32 ± 0.13 in early lactation, from random regression model analyses. Heritability estimates for GFE over the entire lactation were also moderate in primiparous (0.47 ± 0.23) and multiparous (0.43 ± 0.25) cows, with an overall estimate of 0.32 ± 0.13 across all lactations (Spurlock et al. 2012). On the other hand, Köck et al. (2018) reported a low heritability estimate (0.12 ± 0.04) for GFE across all lactations of Austrian Holstein cattle, based on a linear animal model. Spurlock et al. (2012) further observed a strong genetic correlation (0.96 ± 0.18) between GFE in early and mid-lactation. The number of cows used in this study was, however, relatively small (227 and 175, respectively), for multiparous and primiparous cows (Spurlock et al. 2012). In addition, the genetic parameter estimates were based only on the first half of lactation, leaving a gap in knowledge about the latter half of lactation. Although the findings by Spurlock et al. (2012) and Köck et al. (2018) suggest that GFE exhibits sufficient genetic variation to warrant genetic improvement through selection, there is a need to validate these results using larger data sets, and looking at all the stages of lactation.

In an attempt to address the challenge of recording feed efficiency data, several studies have developed models to predict DMI, energy intake and residual feed intake from easy-to-measure traits such as milk production, live weight, mid-infrared spectral data, considering environmental factors such as lactation stage (National Research Council (NRC) 2001; McParland et al. 2014; Shetty et al. 2017; Lahart et al. 2019; Liang et al. 2021). Such models could make it possible to generate large quantities of feed efficiency data, across the whole lactation, at a low cost. Provided they vary genetically, such predicted traits may thus serve as appropriate selection criteria for feed efficiency. Wall et al. (2010) and de Haas et al. (2011) noted that selection on predicted traits can be as effective as selection on the actual trait, particularly when genetic correlations with other traits of economic importance are reasonably strong and records for the predicted trait are available on a large scale. Predicted feed efficiency traits appear to exhibit different genetic variation from the actual measured traits, with varying heritability estimates being obtained among studies (Krattenmacher et al. 2019; Zhang et al. 2020). Moreover, genetic parameter estimates for predicted feed efficiency traits are generally scarce, and their application is still limited.

Madilindi et al. (2022b) developed models to predict daily GFE from live weight and milk components in primiparous South African Holstein cows. Dry matter intake was predicted reliably by a model consisting of only live weight (LW) and milk yield (MY) (*R*^*2*^ = *0.79*; root mean squared error (RMSE) = 1.05 kg/day), while a model that comprised butterfat yield, MY and LW had the highest ability to predict GFE (*R*^*2*^ = *0.98*; RMSE = 0.05) (Madilindi et al. 2022b). This presents a big promise to generate large quantities of data of individual cow DMI and GFE at a low cost, which can be used to implement genetic improvement of feed efficiency. It is, however, essential to first assess the extent to which these predicted traits are under genetic control, and also estimate the genetic parameters for carrying out the requisite genetic evaluation.

The aim of this study was, therefore, to estimate genetic parameters for gross feed efficiency predicted from milk components and its relationship with energy-corrected milk, across the first-three lactations of South African Holstein cattle.

## Materials and Methods

### Data

Test-day records and pedigree data of cows from nine intensively-fed Holstein herds, participating in the South African National Milk Recording and Improvement Scheme, were obtained from the Integrated Registration and Genetic Information System of South Africa (http://www.intergis.agric.za/). Records for the period 2009 to 2019 were considered.

### Energy-corrected milk

Energy-corrected milk (ECM) yield (kg/day) was calculated from test-day milk yield (kg/day), protein (%), butterfat (%) and lactose percent (%), using Eq. 1, according to Kirchgeßner (1997).

1 $$ECM (kg/day)=\mathrm{milk\; yield }\;\left(\mathrm{kg}\right)\times\frac{(0.39\;\times\mathrm{\; butterfat \;\% }\;+\; 0.24\;\times\mathrm{\; protein \;\% }+ 0.17\;\times \mathrm{\;lactose \%}) }{3.17}$$

### Prediction of gross feed efficiency

Prediction models for GFE were developed prior to this study, for primiparous (Madilindi et al. 2022b) and multiparous Holstein cows (Madilindi et al. unpublished). Due to the unavailability of live weight data, the prediction model based on test-day butterfat yield (BFY) (kg/day), which was the third best for primiparous cows, was used to predict GFE in the current study. The prediction model based on test-day BFY (kg/day), which was the best model for multiparous cows, was also used to predict GFE. Equation 2 was used for primiparous cows, whereas Eq. 3 was used for multiparous cows. These models had coefficients of determination (*R*^*2*^) of 0.87 and 0.80, respectively. Gross feed efficiency was calculated as the ratio between ECM (kg/day) and DMI (kg/day).

2 $$pGFE=0.018 + 1.335\times BFY\; (kg/day)$$

3 $$pGFE=0.413+0.759 \times BFY \;(kg/day)$$

### Data preparation and editing

The original data set consisted of 13,332 first to third lactation test-day records of 1,927 Holstein cows. Age of the cow at calving was restricted to the ranges of 20 to 36, 30 to 54 and 40 to 66 months for first, second and third lactation, respectively, so as to exclude outliers (Mostert et al. 2006; Dube et al. 2008). Test-day milk yields of < 3.0 kg or > 99.9 kg, protein percent of < 1% or > 7%, butterfat percent of < 1.5% or > 9%, and lactose percent of < 4.2% or > 5.2% were considered as outliers and excluded. Only test-day records falling between 10 and 305 days in milk (DIM) were included. Each lactation was divided into early (10–100 DIM), mid (101–200 DIM) and late (201–305 DIM) stages. Each animal had a minimum of two ECM and pGFE observations per stage of lactation. The pedigree was built around animals with ECM and pGFE records, and was traced back to four generations. The final data set, after editing, comprised of 11,068 test-day records of 1,575 cows from eight herds. The structure of the final data set used to estimate (co)variance components for ECM and pGFE is presented in Table 1.

**Table 1:** Structure of the data set used to estimate variance components for daily-predicted gross feed efficiency and energy-corrected milk in the first three parities of South African Holstein cattle

Lactation period	Number of Animals	Number of Dams	Number of Sires
Lactation stage			
* Early*	803	688	229
* Mid*	1198	987	300
* Late*	1131	641	286
Entire-lactation			
* Primiparous*	1069	909	251
* Multiparous*	1012	848	257
All lactations	1575	1263	355

### Statistical analysis

Descriptive statistics for pGFE and ECM were calculated using the Proc Means procedure of the Statistical Analysis System (version 9.4, SAS, Institute, Carry, NC, USA). Non-genetic factors influencing pGFE and ECM, which required to be fitted in the models for (co)variance components estimation, were also determined using the General Linear Models procedure of the Statistical Analysis System (version 9.4, SAS, Institute, Carry, NC, USA). These effects included age of cow at calving, lactation stage, parity and herd-test-day.

Bivariate analyses were carried out to estimate (co)variance components, as well as heritability and repeatability, for pGFE and ECM, within stages of lactation, for the entire first lactation (primiparous), entire second and third lactations (multiparous), and all entire lactations pooled together. All the analyses were conducted by the restricted maximum likelihood (REML) procedure of the ASReml 4.2 software (Gilmour et al. 2021). Genetic and phenotypic correlations were estimated for pGFE between stages of lactation and among lactations, as well as between pGFE and ECM within stages of lactation and across lactations. The following repeatability animal models were fitted:

$$\begin{bmatrix}y_1\\y_2\end{bmatrix}=\begin{bmatrix}X_1&0\\0&X_1\end{bmatrix}\;\begin{bmatrix}b_1\\b_2\end{bmatrix}+\begin{bmatrix}Z_1&0\\0&Z_2\end{bmatrix}\;\begin{bmatrix}u_1\\u_2\end{bmatrix}+\begin{bmatrix}W_1&0\\0&W_2\end{bmatrix}\begin{bmatrix}{pe}_1\\{pe}_2\end{bmatrix}+\begin{bmatrix}e_1\\e_2\end{bmatrix}$$

where ${y}_{1}$ and ${y}_{2}$ are vectors of test-day observations for pGFE or ECM; ${X}_{1}$ and ${X}_{2}$ are incidence matrices relating fixed effects to observations; ${b}_{1}$ and ${b}_{2}$ are vectors of fixed effects; ${Z}_{1}$ and ${Z}_{2}$ are incidence matrices relating random animal additive genetic effects to observations; ${u}_{1}$ and ${u}_{2}$ are vectors of animal additive genetic effects; ${W}_{1}$ and ${W}_{2}$ are incidence matrices relating random permanent environmental effects to observations; ${pe}_{1}$ and ${pe}_{2}$ are vectors of permanent environmental effects; ${e}_{1}$ and ${e}_{2}$ are vectors of residual effects.

Animal additive genetic effects $(a)$ were assumed to have the distribution *N* ~ (0, ${A\sigma }_{a}^{2}$), where $A$ is the additive genetic relationship matrix and ${\sigma }_{a}^{2}$ is the animal additive genetic variance. Permanent environmental effects $(pe)$ were assumed to be distributed with *N* ~ (0, *I* ${\sigma }_{pe}^{2}$), where I is an identity matrix, ${\sigma }_{pe}^{2}$ is the variance due to permanent environmental effects and cov $(a, pe$) = 0. Residual effects $(e)$ were assumed to be distributed with *N* ~ (0, *I* ${\sigma }_{e}^{2}$), where I is an identity matrix, ${\sigma }_{e}^{2}$ is the residual variance and cov $(a, e$) = 0.

The (co) variance structure for random effects in the models was as follows:

$$\mathrm{Var}\left[\begin{array}{c}a\\ pe\\ e\end{array}\right]=\left[\begin{array}{ccc}{A\sigma }_{a}^{2}& 0& 0\\ 0& {I\sigma }_{pe}^{2}& 0\\ 0& 0& {I\sigma }_{e}^{2}\end{array}\right]$$

Genetic trend for pGFE, for all the lactations combined, was estimated by plotting average estimated breeding values (EBVs) by year of birth. The EBVs were estimated by the Best Linear Unbiased Prediction method (Henderson 1984) using the ASReml 4.2 (Gilmour et al. 2021).

## Results

### Descriptive statistics

Means and coefficients of variation (CV) for daily pGFE and ECM, by stage of lactation and for entire-lactations, are presented in Table 2. Mean daily pGFE ranged from 1.20 in late lactation to 1.39 in early lactation, with an overall mean of 1.25. Both primiparous and multiparous cows had a mean daily pGFE of 1.26. Mean ECM varied from 25.78 kg/day in late lactation to 29.67 kg/day in early lactation. Multiparous cows produced an average of 2.62 kg/day of ECM more than the primiparous cows, and the overall mean for ECM across lactations was 27.68 kg/day. Predicted GFE showed higher variation in multiparous (CV = 23.02%) than in primiparous (CV = 17.46%) cows. Energy-corrected milk yield was slightly more variable in late (CV = 34.14%) than in early (CV = 28.08%) lactation.

**Table 2:** Summary statistics for daily-predicted gross feed efficiency and energy-corrected milk for stages of lactation and entire-lactations of South African Holstein cows

Items	Traits	Mean	CV (%)
Lactation stage			
* Early*	pGFE	1.39	19.08
	ECM, kg/day	29.67	28.08
* Mid*	pGFE	1.25	20.00
	ECM, kg/day	28.08	29.95
* Late*	pGFE	1.20	21.67
	ECM, kg/day	25.78	34.14
Entire-lactation			
* Primiparous*	pGFE	1.26	17.46
	ECM, kg/day	26.58	29.95
* Multiparous*	pGFE	1.26	23.02
	ECM, kg/day	29.20	31.16
* All lactations*	pGFE	1.25	20.80
	ECM, kg/day	27.68	31.14

*pGFE* predicted gross feed efficiency, *ECM* energy-corrected milk, *CV* coefficient of variation

### Heritability and repeatability estimates

#### Within lactation stage

Estimates of heritability for daily pGFE and ECM within stages of lactation are shown in Table 3. The heritability of pGFE was low to moderate, ranging from 0.09 ± 0.04 in mid lactation to 0.18 ± 0.05 in late lactation. Estimates were low for ECM, varying from 0.12 ± 0.04 in mid lactation to 0.15 ± 0.05 in late lactation.

**Table 3:** Heritability estimates (± se) for daily-predicted gross feed efficiency and energy-corrected milk within stages of lactation in South African Holstein cattle

Lactation stage	pGFE	ECM
Early	0.12 ± 0.06	0.13 ± 0.06
Mid	0.09 ± 0.04	0.12 ± 0.04
Late	0.18 ± 0.05	0.15 ± 0.05

*pGFE* predicted gross feed efficiency, *ECM* energy-corrected milk

#### Entire lactations

Table 4 presents estimates of heritability and repeatability for pGFE and ECM for entire-lactations. The heritability of pGFE was low (0.10 ± 0.04) for multiparous (combined second and third lactations) and moderate (0.21 ± 0.05) for primiparous (first-lactation) cows. Heritability estimates for ECM were also low (0.09 ± 0.04) for multiparous and moderate (0.17 ± 0.05) for primiparous cows. Overall estimates of heritability for pGFE and ECM across all three lactations were low and identical (0.14 ± 0.03). Corresponding estimates of repeatability were mostly moderate, and ranged between 0.42 ± 0.02 for pGFE in multiparous and 0.52 ± 0.02 for ECM in primiparous cows. Repeatability estimates for all the lactations combined were also moderate at 0.37 ± 0.01 and 0.40 ± 0.01 for pGFE and ECM, respectively.

**Table 4:** Heritability and repeatability estimates for predicted gross feed efficiency and energy-corrected milk for entire-lactations of primiparous and multiparous South African Holstein cows

Lactation	pGFE		ECM	
	*h*^*2*^ ± se	*r* ± se	*h*^*2*^ ± se	*r* ± se
First	0.21 ± 0.05	0.45 ± 0.02	0.17 ± 0.05	0.52 ± 0.02
Second and third	0.10 ± 0.04	0.42 ± 0.02	0.09 ± 0.04	0.47 ± 0.02
All	0.14 ± 0.03	0.37 ± 0.01	0.14 ± 0.03	0.40 ± 0.01

*pGFE* predicted gross feed efficiency, *ECM* energy-corrected milk, *h*^*2*^ heritability, *r* repeatability, *se* standard error

### Genetic and phenotypic correlations

#### Correlations for pGFE between stages of lactation

Estimates of genetic and phenotypic correlations among daily pGFE in different stages of lactation are presented in Table 5. Genetic correlations were all positive and high, ranging from 0.87 ± 0.24 between early and mid-lactation to 0.97 ± 0.28 between early and late lactation. Phenotypic correlations were also positive but moderate, varying from 0.40 ± 0.03 between early and late lactation to 0.44 ± 0.02 between mid and late lactation.

**Table 5:** Genetic (upper diagonal) and phenotypic (lower diagonal) correlations for predicted gross feed efficiency between stages of lactation in South African Holstein cattle

Lactations stage	Early	Mid	Late
Early		0.87 ± 0.24	0.97 ± 0.28
Mid	0.42 ± 0.03		0.94 ± 0.12
Late	0.40 ± 0.03	0.44 ± 0.02	

#### Correlations between pGFE in primiparous and multiparous cows

The genetic correlation between pGFE in primiparous and multiparous cows was strong and positive (0.99 ± 0.21). On the other hand, the phenotypic correlation was low (0.27 ± 0.03).

#### Correlations between pGFE and ECM

Table 6 contains genetic correlation estimates between pGFE and ECM within stages of lactation and across all the three lactations. Correlations within stages of lactation were considerably strong and favorable, ranging from 0.90 ± 0.05 in mid lactation to 0.99 ± 0.02 in early lactation. The genetic correlation across all lactations was also high and favourable (0.90 ± 0.03).

**Table 6:** Genetic correlations between daily predicted gross feed efficiency and energy-corrected milk within stages of lactation and across lactations in South African Holstein cattle

Lactation stage	${r}_{g}$± se
Early	0.99 ± 0.02
Mid	0.90 ± 0.05
Late	0.91 ± 0.04
All lactations	0.90 ± 0.03

${r}_{g}$ genetic correlation, *se* standard error

### Genetic trend

**Fig. 1:**
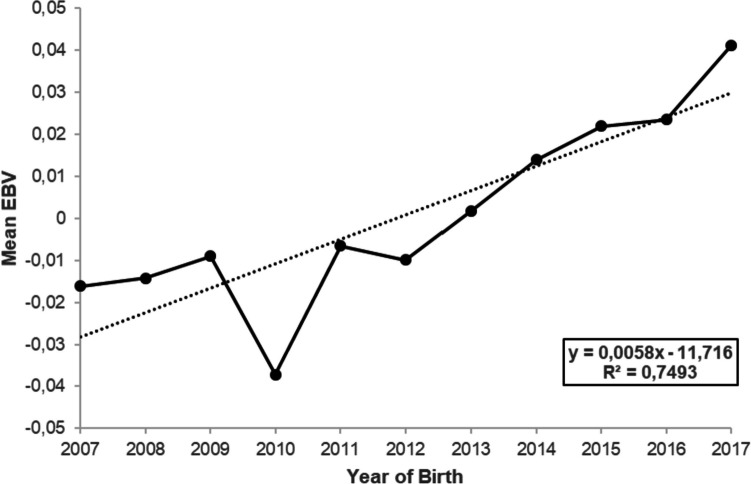
Genetic trend for daily-predicted gross feed efficiency in South African Holstein cattle

Genetic trend for daily pGFE, for cows born between 2007 and 2017, is presented in Fig. 1. There was a marginal increase in mean estimated breeding value (EBV) for daily pGFE from -0.02 in 2007 to 0.04 in 2017, representing a rate of increase of 0.0058 per year during the 10-year period.

## Discussion

### Phenotypic means for pGFE and ECM

Gross feed efficiency (GFE) is an exceptionally important trait in dairy production, due to its impact on profitability and environmental sustainability. Energy-corrected milk is a major predictor trait for GFE, and is also of great economic importance. Besides genetic variation between populations, differences in measurement or prediction methods may account for discrepancies in mean GFE among studies (Köck et al. 2018; Tarekegn et al. 2021; Becker et al. 2022). On the other hand, mean ECM is based on yield of milk, which is invariably measured directly, using standard measuring devices (Kirchgeßner 1997).

Previous studies (Bach et al. 2006; Ishler and Heinrichs 2016) reported relatively higher mean values for actual GFE than those obtained for pGFE in the current study. However, the observation that cows were more efficient in early compared to later stages of lactation concurs with earlier research on intensively-fed Holstein cows elsewhere (Bach et al. 2006; Ishler and Heinrichs 2016). As expected from the normal lactation curve, higher daily yields of ECM were also produced in early than later lactation stages. It has been noted that cows in early lactation are more feed efficient because they mostly utilise their body reserves to derive energy for milk production, which causes an artificial increase in gross feed efficiency (Ishler and Heinrichs 2016; Ledinek et al. 2019). On the other hand, late-lactation cows will be gaining weight; thus lowering their calculated gross feed efficiency. The reduced gross feed efficiency in late lactation should, however, not be viewed negatively because cows need to regain body weight in late lactation, in order to have adequate body reserves for the next lactation. Exceptionally high gross feed efficiency in early lactation may, however, indicate that cows are losing too much weight, which might lead to metabolic disorders (Ishler and Heinrichs 2016; Ledinek et al. 2019).

Mean daily pGFE in first lactation was lower than values for actual GFE observed in other recent studies, despite mean daily ECM yields being comparable (Byskov et al. 2017; Li et al. 2018; Krattenmacher et al. 2019). Although mean daily pGFE was the same (1.26) for primiparous and multiparous cows, multiparous cows produced an average of 2.62 kg/day more ECM. Spurlock et al. (2012) also observed similar means for actual GFE of primiparous and multiparous American Holstein cows. The overall mean for daily pGFE across lactations was lower compared to values reported recently for actual GFE in Austrian, German and Swedish Holstein cattle (Köck et al. 2018; Tarekegn et al. 2021; Becker et al. 2022). Cows in the present study also produced relatively lower daily ECM on average than Austrian, German and Swedish Holstein cattle (Köck et al. 2018; Tarekegn et al. 2021; Becker et al. 2022).

### Heritability estimates for pGFE and ECM

#### Heritability estimates for pGFE

A central objective of the current study was to assess the extent to which pGFE exhibits genetic variation and, hence, determine its suitability as a selection criterion for feed efficiency. The heritability estimates for pGFE within stages of lactation and across lactations were low to moderate, indicating scope for modest genetic improvement through selection. Late lactation had the second highest heritability, with the estimate for early lactation being marginally higher than that for mid lactation. This is consistent with the observation that residual error variance for daily production is lower in late lactation, which results in higher heritability estimates (Meseret and Negussie 2017; Buaban et al. 2020; Wahinya et al. 2020; Tarekegn et al. 2021). Spurlock et al. (2012) also observed moderate albeit larger heritabilities for actual GFE in the first half of lactation, and a higher estimate in early compared to mid-lactation, in a study on American Holstein cattle. In further agreement with Spurlock et al. (2012), heritability was higher in primiparous than multiparous cows, which could be due to the higher residual error variance in multiparous cows. Spurlock et al. (2012) analysed actual measured GFE in the first and second halves of lactation, which may partially explain the disparity in the magnitude of estimates from those of the current study. Additionally, Spurlock et al. (2012) used random regression models, which are better at modelling genetic and environmental variances along the lactation trajectory than repeatability models (Dzomba et al. 2010).

The heritability estimate for pGFE across lactations falls within the range of 0.10 ± 0.03 to 0.18 ± 0.03 obtained for actual GFE in Austrian dairy cattle (Köck et al. 2018). However, since this estimate is comparatively lower than the value observed for the late lactation stage, strategic selection based on measurements recorded only in late lactation may be more effective than considering the entire lactation. Due to the higher heritability of pGFE in primiparous compared to multiparous cows, selection considering first parity records only also appears to be justifiable. Thus, stage of lactation and parity should be taken into consideration when incorporating pGFE in the selection objective.

#### Heritability estimates for ECM

Energy-corrected milk is an important component of the complex feed efficiency trait; hence, knowledge of its genetic attributes is essential to the inclusion of GFE in the selection objective. The heritability of ECM yield has been fairly studied in recent years, mainly based on first lactation records, and most of the estimates obtained were moderate (e.g. Manzanilla-Pech et al. 2014; Li et al. 2018; Krattenmacher et al. 2019). In general, these estimates are larger than the low values obtained in the current study. Manzanilla-Pech et al. (2014) found relatively larger values (> 0.19), with higher estimates in mid compared to early and late lactation, in Dutch Holstein cattle. On the contrary, we observed higher estimates in late than mid and early lactation. Higher heritability estimates, which were larger in early than mid and late lactation, have also been reported in first-parity Holstein populations elsewhere (Li et al. 2018; Krattenmacher et al. 2019). Thus, there appears to be no consistency among studies on the relative magnitude of heritability of ECM by stage of lactation.

Disparities in the heritability of milk production traits between parities is well documented in the literature (e.g. Haile-Mariam and Pryce 2017; Meseret and Negussie 2017; Buaban et al. 2020; Tarekegn et al. 2021). In the current study, we obtained higher heritabilities for ECM in primiparous compared to multiparous cows, which is in agreement with Spurlock et al. (2012). Spurlock et al. (2012), however, found much larger estimates (> 0.24) using random regression models.

A low heritability estimate for ECM across the first three lactations was also observed by Köck et al. (2018) in Austrian Holstein cattle, and suggests selection should be based on parity.

### Genetic correlations and repeatability estimates for pGFE and ECM

#### Genetic correlations for pGFE

Genetic correlations between pGFE in different stages of lactation were estimated to determine if selection applied in one stage will result in improvement along the entire lactation. These correlations were positive and substantially large, supporting earlier findings by Spurlock et al. (2012) who observed a genetic correlation of 0.96 ± 0.18 between early and mid-lactation, for actual GFE in American Holstein cattle. These results suggest that pGFE in different stages of lactation is essentially an expression of the same trait. This further supports the idea to base selection only on data recorded in late lactation.

The genetic correlation between pGFE in primiparous and multiparous cows was also positive and extremely high (close to unity). It means pGFE in primiparous and multiparous cows may be under the influence of the same or linked genes. The observed correlation suggest that selection for pGFE based on first lactation data will result in improvement in later lactations. Such an approach is further justified by our finding that the heritability of pGFE is higher in primiparous than multiparous cows.

#### Correlations between pGFE and ECM

Knowledge of the genetic correlations between pGFE and ECM may assist in improving accuracy of selection of pGFE, as well as incorporating it in the selection objective. These estimates were all positive and substantially high (> 0.90), within stages of lactation and across parities, confirming earlier findings by Köck et al. (2018) who reported strong positive genetic correlations between actual GFE and ECM across lactations in Australian Holstein cattle. Spurlock et al. (2012) also noted that improved GFE was closely associated genetically with increased ECM yield throughout the first half of lactation in American Holstein cattle. These results indicate that the two traits may be under the influence of the same or linked genes, and selection for higher ECM yield is likely to result in a correlated improvement in pGFE. More importantly, accuracy of selection for pGFE can be increased through multiple-trait analysis including ECM.

#### Repeatability estimates for pGFE and ECM

Repeatability was estimated to assess the extent to which repeated measures of pGFE and ECM across lactations are under the influence of permanent effects. Repeatability was moderate (0.37 ± 0.01 to 0.52 ± 0.02) for both pGFE and ECM, in agreement with a previous study by Köck et al. (2018) on Austrian Holstein cattle. Much higher repeatability estimates for ECM (> 0.75) were, however, reported in recent studies on first lactations of Holstein cattle populations elsewhere (Byskov et al. 2017; Krattenmacher et al. 2019). It therefore appears that pGFE and ECM in first lactation is a fairly reliable indicator of performance in later lactations. Thus, culling decisions on pGFE or ECM may be made using only first lactation data.

### Genetic trend for predicted gross feed efficiency

Genetic trend for pGFE, across all lactations, was ascertained to assess if there have been any changes in genetic merit for the trait in recent years, in the South African Holstein cattle population. Such a change may occur as a correlated response to selection for other traits with which it is genetically correlated. There has been sustained genetic selection for yield traits in the South African Holstein cattle population (Ramatsoma et al. 2014), which has unfortunately resulted in a correlated deterioration in functional traits (Banga et al. 2014). The current study found a marginal increase in genetic merit for daily pGFE over the period 2007–2017, which may also be a correlated response to selection for yield traits. This is plausible, given the high positive genetic correlation that we observed between pGFE and ECM. Other researchers (Spurlock et al. 2012; Köck et al. 2018) also noted a correlated genetic improvement of actual GFE due to an increase in milk production traits and a decrease in live weight. Thus, the exclusive focus on selection for yield traits in South African Holstein cattle has, fortunately, not been detrimental to feed efficiency. There is, however, a need to achieve more meaningful genetic improvement of feed efficiency by including it in the breeding objective.

## Conclusion

Results of this study indicate that daily gross feed efficiency predicted from milk components exhibits modest genetic variation, with the highest heritability being in first-parity, and in late lactation. High genetic correlations among pGFE in different stages of lactation indicate that records of pGFE along the lactation trajectory can be considered as repeated measures of the same trait. Hence, selection for pGFE based on late lactation records only seems reasonable, as it would achieve the highest accuracy of selection while improving the trait across the whole lactation. Higher heritability of pGFE in primiparous compared to multiparous cows, coupled with high genetic correlations between these lactations, justifies selection on first lactation records only. There appears to be scope for improving accuracy of selection for feed efficiency through multiple-trait analysis including ECM, due to the high genetic correlation between the two traits. Genetic trends show a slight increase in the genetic merit for pGFE in South African Holstein cattle in recent years, which may be a correlated response to selection for higher milk yield or other correlated traits. There is, however, a need to achieve significant improvement in the genetic merit of feed efficiency, in order to achieve profitable and environmentally sustainable dairy production systems. The genetic parameters obtained in the current study can be applied to estimate EBVs for pGFE, which may be used to achieve such improvement. Further enhancements to the selection programme could be effected through the application of random regression modelling, as well as identification of markers or genes influencing feed efficiency.

## Data Availability

The datasets generated during and/or analysed during the current study are available from the corresponding author on reasonable request.
